# Insect Artifacts Are More than Just Altered Bloodstains

**DOI:** 10.3390/insects8020037

**Published:** 2017-03-28

**Authors:** David Rivers, Theresa Geiman

**Affiliations:** Department of Biology, Loyola University Maryland, Baltimore, MD 21210, USA; tmgeiman@loyola.edu

**Keywords:** forensic entomology, insect artifacts, fly spots, blow flies, calliphorids, sarcophagids, bloodstain evidence, crime scene investigation

## Abstract

The bases for forensic entomology are that insects and their arthropod relatives can serve as evidence in criminal, medical and civil legal matters. However, some of the very same species that provide utility to legal investigations can also complicate crime scenes by distorting existing body fluid evidence (e.g., bloodstains, semen, saliva) and/or depositing artifacts derived from the insect alimentary canal at primary or secondary crime scenes. The insect contaminants are referred to as insect stains, artifacts, specks or spots, and are most commonly associated with human bloodstains. This review will discuss the different types of insect artifacts that have been described from crime scenes and laboratory experiments, as well as examine insect contaminates (non-blood based artifacts, transfer patterns, meconium, and larval fluids) that have received little research or case attention. Methods currently used for distinguishing insect stains from human body fluids will also be discussed and compared to presumptive tests used for identification of human body fluids. Since all available methods have severe limitations, areas of new research will be identified for the purpose of development of diagnostic techniques for detection of insect artifacts.

## 1. Introduction

Insects do not commit crimes, but they can be instrumental in solving them. This is especially true with necrophagous Diptera that are attracted to human remains. Foraging adults from several families of flies seek out a corpse as a source of nutrients for egg provisioning and oviposition/larviposition [1]. For these same species, larval nutriment is largely derived from the remains, establishing a linkage between immature development, the corpse, and ambient conditions. With such information in hand, an estimate of the minimum post mortem interval (PMI_min_) can be made [2]. This is considered the primary focus of forensic entomology within the subdiscipline of medicocriminal entomology [3]. The utility of insects to criminal investigations is not limited, however, to time estimates and may include determination of whether a corpse has been moved from another location inferred from faunal, developmental or seasonal information [4]; detection of illicit or prescribed medications bioaccumulated within insects that fed on the corpse [5]; person’s identification based upon DNA profiling of tissues consumed by necrophagous insects [6]; and discovery of gunshot or bomb residues in larval tissues when otherwise undetectable in human remains [7]. Though more obscure, adult flies can also be a source of DNA from imbibed body fluids, which are then deposited as artifacts in other locations, the latter being especially important if the primary crime scene has already been cleaned following body removal [6].

In contrast to their roles as physical and trace evidence, necrophagous insects are not always helpful at crime scenes. The foraging activity of necrophagous Diptera has the potential to be counterproductive to criminal investigations, largely as a result of the way they feed. For flies in the families Calliphoridae and Sarcophagidae, adults land on or beside a corpse, walking across the surfaces or through wet body fluids. Gustatory receptors located at the tips of tarsi (pulvilli) and on the sponging mouthparts are used to assess the nutritional value of the fluids and tissues. Applying Locard’s Exchange Principle to the interaction between necrophagous flies and a corpse, evidence of this association will be left behind at the crime scene [8]. Foraging activity is known to cause mechanical disruption of pooled blood and body fluid stains that have not dried [9,10]. It can also lead to transfer patterns, created by tarsi or the abdomen leaving impressions after passing through wet fluids, either at the primary scene or at other sites [11,12]. As adult flies consume body fluids, they regurgitate and defecate some of the ingested food onto surfaces at or near the crime scene, creating unique stains and/or an intermixing of fly artifacts with bloodstains and other human body fluids [11,13]. Fly contaminates are not restricted to the primary crime scene, as adults display positive phototaxis, and thus are attracted to windows and lights, locations in which wet blood may be transferred or artifacts deposited. In essence, false secondary crime scenes are established as a direct consequence of foraging activity on a corpse.

The problems with fly artifacts are magnified by the fact that regurgitate and defecate are virtually indistinguishable from human bloodstains. Fly stains are morphologically very similar to impact (i.e., forward, back, and mist-like spatter), projected, sneezed, and expirated bloodstains [10], and cannot be reliably distinguished using presumptive or confirmatory tests available for identification of human blood [13,14,15]. The use of molecular methods, namely DNA typing, for person’s identification does not overcome these limitations since complete DNA profiles can be obtained of an individual from blood consumed by flies [6,16]. A few methods have been reported to be useful in differentiating fly artifacts from human bloodstains [9,10,13,17], but all have limitations that prevent each from being consistently reliable for use in crime scene investigations. The reality is that artifacts from very few fly species have been examined to come to any consensus on the typical classification of regurgitate and fecal stains, or accurate methods of detection.

In this review, a discussion of the different types of insect artifacts that have been described from crime scenes and laboratory experiments will be presented, along with an examination of insect contaminates (non-blood based artifacts, transfer patterns, meconium, and larval fluids) that have received little research or case attention. Methods currently used for distinguishing insect stains from human body fluids will also be discussed and compared to presumptive tests used for identification of human body fluids. All available methods have severe limitations, and thus, new areas of research will be identified for the purpose of development of diagnostic techniques for detection of insect artifacts.

## 2. Insect Artifacts

In theory any insect that interacts with a corpse or associated exuded body fluids can potentially create artifacts that confuse reconstruction efforts at a crime scene. However, the reality is that several species of necrophagous Diptera are the chief culprits in producing insect artifacts [11]. As a consequence, this review will focus on stains that result from the activity of flies in the families Calliphoridae and Sarcophagidae, which coincidentally also correspond to the vast majority of the literature focused on insect artifacts. By definition, only one type of insect artifact is officially recognized by bloodstain pattern analysts: insect stains. The Scientific Working Group for Bloodstain Pattern Analysis (SWGSTAIN) has defined insect stains as those bloodstains produced as a result of insect activity [18]. This definition leaves open the possibility of producing insect stains by two methods: insect modification of existing bloodstains or creation of new stains. It is the latter that is most frequently cited by forensic entomologists, since both regurgitation and fecal elimination can yield insect stains containing human blood. The reality is that necrophagous flies can produce stains or artifacts as a result of feeding on several types of fluids (e.g., blood, saliva, semen, vaginal fluids, decomposition fluids), and which yield artifacts that vary widely in terms of shape, color, and size [12,14,19,20]. Deposition of artifacts is also not restricted to just foraging adults, as post-feeding larvae and newly emerged adults have the potential to contaminate crime scenes with unique artifacts. The small literature base that exists for fly artifacts is predominantly focused on regurgitate and defecatory stains, which arguably are the most frequently encountered at crime scenes and most likely to compromise bloodstain pattern analysis. What follows is a brief description of five types of fly artifacts (regurgitate, defecatory stains, transfer patterns, meconium, and larval stains) that have been identified or could potentially be found at crime scenes, including the mechanisms used to produce each type and predicted chemical composition of the stains (Table 1).

### 2.1. Regurgitate

**Figure 1:**
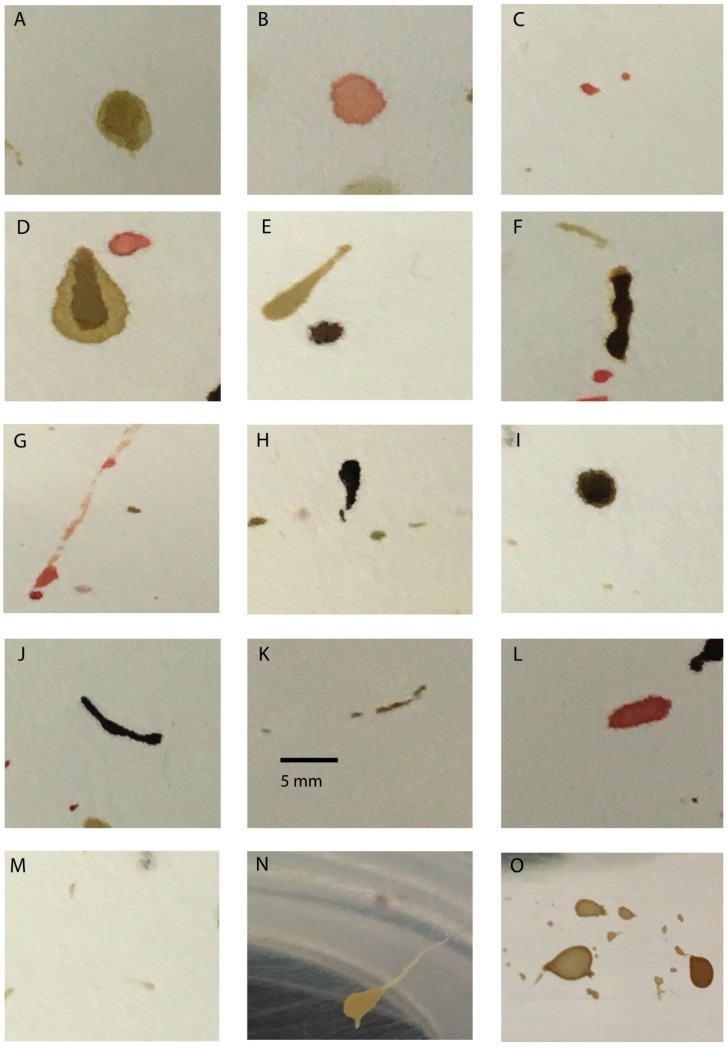
Types of fly artifacts produced by adult calliphorids and sarcophagids. All artifacts were produced following feeding or exposure to human blood, unless otherwise noted. Regurgitate stains deposited by (**A**,**D**,**E**) *Sarcophaga bullata* (Sarcophagdae), (**B**) *Calliphora vicina* (Calliphoridae), (**C**) *Lucilia sericata* (Calliphoridae); defecatory stains deposited by (**F**,**G**) *S. bullata*, (**H**,**I**) *C. vicina*; translocation stains deposited by (**J**,**K**) *S. bullata*, (**L**) *C. vicina*; tarsal tracks produced by *S. bullata*; and meconium deposited by (**N**) *S. bullata*, (**O**) *C. vicina*. Images were captured using a ChemiDoc Imaging System (BioRad).

Regurgitation is considered to be the expulsion of food from any location within the foregut out the oral opening [21]. It is a component of bubbling behavior that leads to a food droplet or bubble forming on the distal tip of the pseudotrachea. Regurgitation is in contrast to vomiting, in which food from the midgut is forced into the foregut, and then passed out of the mouth. Distinguishing between the two would seem to have little forensic value, but in fact the importance lies in composition differences, which can be the bases for development of confirmatory tools used to detect fly artifacts. Additionally, at times the terms have been used interchangeably in the forensic entomology literature [20,22], but clearly the two are separate physiological processes. Regurgitate stains should be considered far more significant as potential contaminants at crime scenes, since bubbling behavior is quite common among necrophagous Diptera, especially following consumption of a meal. After imbibing a liquid diet, the ingested food is pushed through the anterior foregut to the crop. The crop serves as the initial site of mixing of food with salivary enzymes [21]. If the crop was already full when the meal was ingested, regurgitation of the crop contents occurs [23]. This is manifested as bubble formation at the tips of the mouthparts. The bubble itself is highly variable in color, presumably a reflection of the food consumed. That said food bubbles might appear clear despite the recent consumption of carrion, blood, or feces [11] (Figure 1).

The fate of the regurgitated droplet appears to be dependent on the composition of the meal consumed, fly species, and whether the adult is disturbed during bubbling. Bubbling behavior is thought to serve two primary functions; decrease water content of the food prior to enzymatic digestion or flight, and to permit extra-oral digestion [13,21]. It is the latter function that causes regurgitation stains to achieve status as trace evidence at crime scenes. Adult flies will simply drop the food bubbles from the mouthparts onto the substrate they are resting. This can occur around a corpse following feeding on tissues, decomposition fluids, blood, or any other type of exposed body fluid, with the exception of urine [19]. Regurgitate stains are typically round or asymmetrically round (i.e., elliptical or oval) owing to the food bubbles dropping essentially perpendicular to the substrate [12], although occasional tear-drop shaped artifacts have been reported for *Calliphora vicina*, *Sarcophaga bullata*, *Lucilia sericata*, and *Chrysomya megacephala* [6,10,12,17,20] and the tails appear to result from flies beginning to move before release of the droplet [12]. A third morphological pattern reported is dome shaped craters that supposedly result from the sucking process of fly mouthparts [24]. This would seem to implicate regurgitate stains, since adult flies are not known to consume their own feces. However, the idea that the hydrostatic action of the cibarial pump and associated muscles of the foregut can create sufficient negative pressure to suck a stain from a surface or modify dry fly artifacts has not been demonstrated experimentally. In fact, cratered fly stains have only been reported for two species of calliphorids (*Ch. megacephala* and *L. cuprina*), both under laboratory conditions [19,20], but with *L. cuprina*, the stain morphology was attributed to drying on a smooth, non-porous surface, and not due to feeding activity of adult flies. This type of stain is not commonly encountered [25].

Regurgitate stains are composed predominantly of the meal consumed. However, the food is modified by the addition of salivary enzymes that are mixed into the gut fluids within the crop. In adult *Protophormia terraenovae*, enzymes consistent with trypsin, chymotrypsin and pepsin have been detected in the crop and exogenously deposited regurgitate [26]. Presumably a similar enzyme profile would be expected in other carrion-inhabiting calliphorids, although these same proteases have not been observed in *Phormia regina* [21]. Interesting, *P. regina* apparently does not typically drop bubbles and is more apt to reabsorb the regurgitated food [23]. When regurgitate is released by this species, it is twice as likely to occur following a protein meal than after sugar consumption [23]. By contrast, most other species of calliphorids observed bubbling do release regurgitate [24], and the event coincides with feeding on tissues and fluids from human remains or carrion. In other words, diets high in protein [1]. It is also quite likely that antimicrobial compounds produced by the labellar glands are released into the ingested food since uptake of bacteria from decomposing food sources is inevitable [21,27]. Endogenous bacteria and possibly other microorganisms conceivably are present in the crop of necrophagous Diptera, or are introduced to ingested food if vomiting occurs to permit intermixing of enzymes from the midgut. Regardless of the precise composition, stains resulting from regurgitated food are expected to be chemically similar yet distinct from the original food source.

The discussion of regurgitate (or any other insect artifact) composition is based on the assumption that adult flies consume fluids and tissues found at the crime scene. In reality, many flies may be introduced to the corpse during the crime scene investigation; that is, the adults gained access as a result of doors, containers, etc. being opened by the first responders to the scene [11]. In such scenarios, deposition of regurgitate and/or feces chemically distinct from fluids or tissues of the deceased may be introduced at the crime scene. This includes the possibility of fly artifacts containing DNA from an individual not associated with the current crime scene.

### 2.2. Defecatory Stains

Defecatory stains result from adult flies eliminating liquid feces. These stains are varied in terms of shape and color based on species. Eliminated feces on smooth surfaces commonly appear round or asymmetrically round, sometimes with tails, and on occasion may appear elongate (linear or sausage shaped) [12,19,20]. In most cases, if a fly artifact possesses a tail, the stain is derived from feces. The tail originates from either the fly beginning to walk prior to completion of defecation, producing a tail tapered in the direction of fly movement, or results from forcible expulsion of liquid feces from a protruded anus. The latter also yields tails pointed in the direction of fecal droplet movement. In theory, artifacts with tails permit distinction between defecate and regurgitate, and between bloodstains and defecatory stains. The idea is based on the premise that defecatory stains are often formed into shapes—sperm-shape, tadpole, or tear-drop—unique from other types of stains found at crime scenes [11,19]. However, as will be discussed later in this review, the uniqueness between these types of stains is not quite as distinct as originally thought.

Stains resulting from fecal release are the most commonly produced artifacts by *L. cuprina* and *C. vicina* (collected in Frankfurt, Germany) following feeding on human blood [6,14]. In contrast, regurgitate stains are the most abundant artifacts released by *S. bullata*, *L. sericata*, *Ch. rufifacies*, *Ch*. *megacephala*, and *P. regina* following consumption of a range of foods, including human blood [10,12,17,20]. Interestingly, Rivers and McGregor [12] demonstrated that *C. vicina* collected in Baltimore, Maryland predominantly deposits regurgitate stains, and that nearly all types of artifacts produced by this fly are round and lack tails. The latter differs from the observations of Striman et al. [17], in which *C. vicina* obtained in Nebraska frequently deposited defecatory stains that were tear-drop shaped with long tails. Durdle et al. [19] reported that defecatory stains of *L. cuprina* are more varied in shapes, colors, textures, viscosity, and translucence than observed with any other fly species. Varied fecal stain morphologies have also been reported for *Ch. megacephala* collected in Malaysia, in which tear-drop, sperm-like, snake-like, and irregular tadpole-like defecate with long tails (4.8 to 9.2 mm) were produced on porous surfaces following *ad libitum* consumption of chicken blood [20]. Such differences in regurgitate and defecatory artifact morphology appears to reflect species and diet dependency, as well as within species variation based on geographic location [12].

Fly feces is a unique mixture of partially and undigested food, and various metabolic wastes deposited from the Malpighian tubules and hindgut [28]. Defecate contains relatively high concentrations of ammonia, allantoin/allantoic acid, and uric acid [29], all of which are absent from regurgitate. Several proteolytic enzymes (e.g., pepsin, trypsin, α-glucosidase, β-galactosidase, β-glucosidase, amylase, fructofuranosidase) released by the midgut epithelia can also be found in feces [30,31]. Enzyme type, quantity, and level of activity are dependent on food composition and most likely fly species. Like with the adult crop, endogenous bacteria and other microorganisms are present in the hindgut, and possibly exogenously acquired microorganisms, presuming that they can survive the highly acid environment of the midgut [29]. Defecatory stains are expected to be chemically similar yet distinct from regurgitate and the original food source. That said Weiss [32] states that it is possible in certain instances for ingested food to pass through the alimentary canal unmodified. In which case, feces, regurgitate, and the original food source may be virtually indistinguishable from each other based on chemical composition. Variable degrees of food processing by an adult fly may also account in part for the wide variation in physical features of defecatory stains.

### 2.3. Transfer Patterns

Transfer patterns are defined as bloodstain patterns resulting from contact between a wet bloody object (surface) with that of another object or surface [18]. This definition can be extended to that of insects that interact with a corpse or exuded body fluids. For example, adult flies can generate transfer patterns by dragging the abdomen through or across food and then transferring a wet impression to another location. This form of transfer pattern is referred to as translocation [12], and is known to occur with adult flies, cockroaches and ants that frequent crime scenes [11]. Translocation stains typically are asymmetrical linear stains and appear the same color as the food source that was transferred by the insect’s body. A second type of transfer artifact is called tarsal tracks. These transfer patterns are formed by adult flies or other insects walking through wet food and leaving impressions of tarsi or pulvilli on another surface. Tarsal tracks produced by adult flies are typically small (<0.2 mm in diameter), round stains that appear the same color as the food source walked through by the insect [12]. These fly footprints are often randomly distributed across the surface of an object but have also been shown to occur in clusters with some species [12]. Since both translocation stains and tarsal tracks are produced from simply walking through or across a food source, the composition of the artifacts will be identical to the original source. It should also be noted that the behaviors of flies and other insects that lead to transfer patterns also alters the morphology of existing stains (wipe patterns) and insect artifacts [19].

### 2.4. Meconium

Meconium is a creamy, viscous fluid released via the anus of newly emerged adult flies [22]. It is produced during intrapuparial development and represents metabolic waste products that accumulate in the partially formed adult gut. Meconium is thus a form of storage excretion since waste removal is not possible until after the adult emerges from the puparium [28]. As a consequence, storage of nitrogenous end products occurs in non-toxic forms, predominantly as uric acid and allantoin [33], which in turn are the major components of excreted meconium. The presence of uric acid is also largely responsible for the creamy white to yellow coloration of the deposited fluid.

The occurrence of meconium at a crime scene is indicative of a long association between the fly and the remains, since all phases of fly development outside of adult maturation have occurred at that location or close by. Meconium is deposited within minutes of emergence from the puparia but prior to expansion [22]. What this means is that adult flies have yet to gulp air into the crop, so the wings appear shriveled and the abdomen narrow. Consequently, meconium is usually deposited on the substrate near the site of extrication and would be less commonly found on walls or furniture. The size and shape of meconium stains vary based on species but are typically large (>2 mm in diameter), round to asymmetrically round (ellipse or oval) stains that often have long tails. Like with defecatory stains, the tails result from the fly beginning to walk prior to completion of elimination, and thus point in the direction of fly movement.

### 2.5. Larval Stains

Necrophagous flies deposit unique forms of artifacts at crime scenes that are readily distinguishable from all other forms: eggs and larvae. Juvenile forms of Diptera are never referred to as contaminates or artifacts but they do potentially confound crime scene investigations in at least two ways: through consumption of physical evidence, including the corpse, and by modification of existing stains via transference or deposition of larval fluids. Larval stains generally would not be expected to be confused for human bloodstains or other body fluids, but the activity of maggots potentially can alter the physical appearance and/or chemical composition of existing stains. The latter obviously has the potential to compromise the utility of presumptive or confirmatory tests performed during an investigation. Here, the focus is on fluids derived from larvae since the chemical composition of each type is unique from human body fluids. Two types of larval-derived fluids will be considered, secretions from the oral opening and excretions released from the hindgut via the anus.

#### 2.5.1. Secretions

Larvae from the families Calliphoridae and Sarcophagidae, and to a lesser extent Muscidae, feed on human remains and carrion in larval feeding aggregations or maggot masses [34]. For most species, the aggregations form during the late second to early third stage of larval development. Within the feeding masses, larvae release oral secretions on to the food substrate to promote extra-oral digestion [34]. The food substrate obviously can be human remains, body fluids or stains, and feces. Thus the potential exists for modification of trace evidence by larval secretions. The composition of the secretions is the key to this potential for alteration. Larval salivary glands produce amylase, invertase, proteases, and hydrolases [31], with chymotrypsin functioning as the major orally secreted protease in larvae of *L. cuprina* [35]. Several other species, including *L. sericata*, *C. erythrocephala*, and *Sarconesiopsis magellanica*, release a cocktail of proteases (e.g., trypsin-like, chymotrypsin-like, leucine aminopeptidase, aspartyl proteinase, metalloproteinase, and carboxypeptidase A and B) in secretions that promote digestion of necrotic tissues and other food sources [36,37,38]. Larvae of *L. sericata* also secrete collagenase, although the source of the enzyme may be the midgut rather than salivary glands [39]. Larval secretions also contain a range of antimicrobial compounds [40,41,42], however, only the peptides lucifensin and lucifensin II have been identified thus far [43,44].

Like with meconium, the presence of larval secretory stains suggests a long association with the corpse as only older (late 2nd or 3rd instars) larvae can survive any significant length of time off the remains before returning to feed [45,46]. Such stains are also most likely to occur on non-porous surfaces like tile, wood, or vinyl floors since carpets and fabrics are potentially desiccating conditions to any age larva.

#### 2.5.2. Excretions

Larval feces are broadly comparable to the composition of adult defecate. In practical terms, this means larval feces contains partially and undigested food, various metabolic wastes, digestive enzymes, endogenous bacteria, and the remains of lysed or digested exogenously acquired bacteria [28,47]. Larvae excrete high concentrations of ammonia, nonionic ammonia and allantoin throughout the feeding stages [29,48] and especially when purging the gut prior to pupariation [49,50]. Other nitrogenous products (i.e., uric acid and allantoic acid) are present in feces but in lower concentrations [28,33]. An array of digestive enzymes is released into the lumen of the midgut, including trypsin, chymotrypsin, pepsin, collagenase, lysozyme, β-galactosidase, α-glucosidase, fructofuranosidase, maltase, amylase, and lipases [31,51,52,53], with the potential to pass out of the gut in feces. Indeed, excretions of *L. cuprina* contain several proteases, with chymotrypsin being the most abundant [35]. However, in larvae of *Musca domestica*, only 20% of the enzymes are released in excreta, as an endo-ectoperitrophic circulation in the midgut facilitates enzyme recovery before entering the hindgut [54]. Trypsin and amylase are the most prevalent hydrolases in excretions of *M. domestica* [54], but neither are distinctive from human fluids based on enzymatic activity alone. Larval defecate is also comprised of a number of antimicrobial agents including small peptides (lucifensin and lucifensin II), urea, phenylacetic acid, phenylacetaldehyde, and calcium carbonate [42,43,44].

It is during the postfeeding stage of larval development in which most species will wander from the food to seek refuge to initiate pupariation [50]. Larvae will disperse from the food source and initiate random crawling, during which time the immatures will migrate through existing fluids or stains in their path. This behavior not only distorts stain morphology (wipe pattern) but also alters the chemical composition as transfer of fluids adhering to their body occurs, as does release of excreta from the anal opening. Larval trails are evident from human remains across non-porous surfaces, revealing the initial path of travel of the maggots.

## 3. Methods of Detection

A variety of methods have been purported to permit some differentiation of fly artifacts from human bloodstains. While there is limited success with each technique, at present, no empirical methods exist for reliable distinction between insect-derived artifacts and body fluids. Langer and Illes [27] reviewed the techniques available for identifying insect artifacts at crime scenes, as well as delineating the limitations of each method. Since no new developments in methodology have occurred since their review, we will not attempt to duplicate their efforts here. Instead, a brief description of each method will be presented to provide context for areas in need of new research. The methodology is grouped according to the categories proposed by Langer and Illes [27]: visual, contextual, and chemical methods of detection.

### 3.1. Visual Methods

Visual methods rely on comparative morphology and alternate light detection. The morphological features of fly artifacts, especially because they may be construed as irregular shaped by comparison to human bloodstains, are believed by some to be distinctive, and thus would not be confused by a trained expert in bloodstain pattern analysis with true bloodstains [19]. While this is undoubtedly true in some scenarios, many crime scene analysts do not have a background in forensic entomology to readily recognize fly artifacts [6]. Importantly, subjective analysis is not a satisfactory means for distinguishing fly artifacts from other forms of trace evidence for a number of reasons. For one, the morphology of regurgitate can be distinct from that of defecatory stains for some species [13,19,20], but not others [10,12,17]. A semi-quantifiable method has been proposed as one means of visually identifying fecal stains [13]. The method depends on the ratio of the length of stain tail to the length of stain body, which if greater than one, supposedly excludes bloodstains. Thus, the method relies on a process of elimination of stain suspects to identity defecatory stains. One problem with this approach is that the ratios generated to evaluate this technique are from limited sample sizes at crime scenes and from laboratory tests that did not utilize blood as a food source for the flies, and only one species of fly was tested for validation [13]. However, the authors have reported using the technique many times since and remain convinced of its validity in recognizing potential defecatory stains [25]. A second concern is that for several species, tails are commonly absent from defecatory spots, yielding fecal and regurgitate stains that are indistinguishable from each other [10,12,17]. A third issue is that the underlying premise that a tail length to body ratio exceeding one excludes all forms of human bloodstains is not correct [55]. In reality, fly artifacts are highly variable in size, color, and morphology due to unique species behaviors, size of blood meal, and time taken to consume the meal, as well as being dependent on the physical surfaces on which they have been deposited [19].

A second method of visual identification of fly artifacts involves the use of alternate light detection. The method has been used with only two species (*C. vicina* and *L. sericata*), in which expelled defecate could be visualized by alternate lighting at 465 nm with an orange contrast filter [9,10]. The authors indicate that neither regurgitate or human blood fluoresced under the same conditions. However, this method is not entirely satisfactory since in the absence of tails and depending on fly species, regurgitate and defecatory stains can be very difficult to distinguish from each other based on morphology [6,17]. The alternate light technique also does not make a distinction between other forms of body fluids that may be present at a crime scene, and there have been no reports as to whether the method is effective at detection of translocation stains or tarsal tracks. Also lacking is an understanding of the mechanisms that lead to fluorescence of defecatory but not regurgitate stains and human blood. Fujikawa et al. [10] speculate that the presence of urea in defecate accounts for detection at 465 nm. This seems unlikely in that only trace amounts of urea are present in adult excreta [28,33], human blood contains small quantities of urea yet did not fluoresce [10], and other body fluids that do not contain nitrogenous end products can be detected by the same technique [8].

### 3.2. Contextual Methods

Contextual methods of fly artifact detection rely on visual analysis coupled with where the insect stains occur in relation to one another and with respect to human body fluid stains [27]. There are two ways to consider contextual analysis of suspect stains: one is to examine the stains based on physical location with respect to the crime scene or other stains, and the other is to compare the directionality of multiple stains located in close proximity. The former is relevant to stains being located in seemingly unusual or atypical circumstances by comparison to other trace evidence. For example, adult flies display positive phototaxis, so they are attracted to artificial and natural light sources. As a consequence, fly artifacts may be deposited on lampshades, light fixtures, windows, curtains, window shades, windows casings, and other objects in close proximity to the light sources [11]. The net effect can be that stains consistent (e.g., morphology and/or chemistry) with human blood are present in locations not consistent with other physical or trace evidence associated with a crime scene or pathology reports [27]. Necrophagous flies will also forage for other food sources, most prominently located in kitchen and food storage areas. Again, fly artifacts may be deposited in these locations, which would be especially suspicious if no other evidence is found in such areas. Despite the irregularity of locations, trained bloodstain analysts only can rely on subjective determination to include or exclude these stains in their analysis.

When multiple stains are present with some showing directionality not consistent with other stains, the possibility exists that insect artifacts are intermixed with human bloodstains [27]. An alternative explanation is that cast off patterns are present from multiple impacts or trauma events. In the latter scenario, multiple patterned bloodstains are recognizable to trained bloodstain analysts who could group the stains based on consistent directional patterns and angles of impact [56]. However, when insect artifacts are intermixed with castoff or impact bloodstains, the insect-derived stains can only be identified based on discretionary interpretation by individual analysts. Fly activity may be suspected based on the presence of stains with random directionality, round stains intermixed with bloodstains with tails that can be grouped based on consistent directionality, or by the occurrence of irregular shaped stains typical of translocation, defecatory stains with distinctive tails (i.e., sperm-shaped, tadpole shaped, tear-drop shaped) or dome shaped craters. Thus, visual analysis is used to detect irregular or atypical stains in relation to other stains or evidence found at the scene. At present, there is no means to confirm that suspected fly artifacts are in fact derived from necrophagous flies or other insects.

### 3.3. Chemical Methods

Chemical analyses in the form of presumptive and confirmatory tests provide the most definitive evidence that fly artifacts cannot be reliably distinguished from human bloodstains or other body fluids. This assertion is based on the idea that fly artifacts are the direct result of adult flies’ interaction with a corpse and/or associated body fluids. The most obvious examples are regurgitate and defecatory stains produced after consumption of human blood. Presumptive chemical tests designed to detect animal blood will test positive for both fly artifacts and true human bloodstains [9,10,13]. This is true regardless of the type of presumptive blood test employed. That said Durdle et al. [15] demonstrated that some differentiation could be made with presumptive tests used on artifacts (regurgitate and defecate) produced by adults of *L. cuprina* following feeding on human blood or semen if the stains were relatively fresh (3-days-old) but not if 2-weeks-old nor if flies consumed saliva. Confirmatory tests for semen and saliva demonstrated similar trends in that fresh but not older artifacts could be distinguished from the original food sources. Confirmatory tests designed for human blood do not permit distinction between fly artifacts and bloodstains, and DNA typing does not overcome this limitation since complete DNA profiles of the victim or offender have been obtained from regurgitate and defecatory stains [6,16]. Despite some limited differentiation using chemical analysis, Durdle et al. [15] concluded that presumptive and confirmatory testing could not be used reliably to detect fly artifacts. However, in combination with visual analysis, the utility of chemical analysis may potentially improve.

Translocation and tarsal tracks have not been tested by chemical analysis, but based on the mechanism of production, should not be distinctive from the original food source. Necrophagous flies generally do not consume saliva and semen found at crime scenes, and even when they are fed upon, the resulting artifacts are often difficult to visualize [6]. The latter poses a potential serious problem since fly artifacts derived from such fluids contain human DNA and thus are a source of extraneous DNA whose origin would be unknown since detection of the stain is limited.

## 4. New Research

The inability to consistently and reliably distinguish insect artifacts from human bloodstains and other body fluids represents the biggest issue with respect to entomological contaminants at crime scenes. There has been modest success with a few methods designed for visual, contextual, or chemical analysis of fly artifacts, but none are satisfactory based on several limitations. The deficiencies include a lack of reliability, no single technique is suitable for all fly species, none make a distinction from other forms of body fluids that may also be present at crime scenes, all are presumptive not confirmatory tests, assessment of artifact morphology is dependent on a very small pool of forensic experts, and very few forensically important species known worldwide have been examined by the reported methods for discerning fly artifacts from human bloodstains and other bodily fluids. The latter makes it very difficult to come to any consensus on the typical classification of fly artifacts or accurate methods of detection. Durdle et al. [19] suggested that the use of two methods in conjunction with one another (i.e., presumptive blood testing coupled with visual analysis) may improve the precision in distinguishing fly artifacts from human bloodstains. Nonetheless, the techniques alone or in combination still should be viewed as inconsistent and non-quantifiable, especially in terms of the visual analysis component. A lack of diagnostic tests for the identification of insect artifacts at a crime scene means that only subjective interpretation is currently used to distinguish fly evidence from bloodstains. The take home message is that at present, insufficient data and methodology are available to make consistently reliable and quantifiable distinctions between insect artifacts and stains from human body fluids. This obviously means that new research is needed to overcome the stated deficiencies and develop new methods for detection of fly artifacts.

### 4.1. Chemical Analysis Based on Composition of Fly Fluids

The majority of presumptive and chemical tests available for forensic serological analysis at crime scenes relies on chemical testing. In the context of human body fluid stains, discernment is based on unique chemical properties of each type of fluid that minimally leads to broad classification. Confirmatory tests, especially in the cases of human blood, semen, and saliva, depend on immunoassays employing polyclonal or monoclonal antibodies that recognize unique human-specific antigens [57]. In this respect, very little research has been done to analyze the chemical composition of fly artifacts, aside from DNA analysis for the purpose of identifying the source of blood, or inferences made based on the results of presumptive blood tests [6,9,10,13]. Rivers et al. [26] demonstrated that regurgitate stains deposited by *P. terraenovae* possess at least three (trypsin-like, chymotrypsin-like, and pepsin-like) digestive enzymes that were also found in the crop of the adult fly, independent of the food source. These observations point to further research into the potential of fly digestive enzymes as the bases for new chemical methods of detection of insect artifacts. Most likely presumptive tests in the form of enzymatic assays would not be sufficient to distinguish artifacts from human body fluids, as many fly enzymes overlap in substrate specificity with vertebrate enzymes [31]. However, in several instances, though enzyme functionality is similar to vertebrate counterparts, enzyme structure is substantially different [58,59,60]. This is especially true for the various forms of trypsin (i.e., earlier, late) that are produced in the midgut of several Diptera [61]. Consequently, fly digestive enzymes, potentially from all feeding stages that interact with human remains and body fluids, could be used as antigens for the development of immunological tools used in confirmatory tests. Similarly, the small antimicrobial peptides lucifensin and lucifensin II are potential candidates for antibody development to use in immunoassays that recognize larval stains or distortions of existing human body fluid stains caused by maggot activity. Further research is needed to determine if adult labellar glands synthesize these peptides as well, and if so, this would greatly increase the utility of the peptides in development of immunological diagnostic tools.

Two potential limitations of any of the described potential antigens are that (1) they likely would not be able to distinguish regurgitate from defecatory stains; and (2) would also not recognize translocation and tarsal tracks as being separate stains from the original source, meaning human body fluids. In terms of the former, this should be viewed as a minor weakness since the primary need is to differentiate fly artifacts from human bloodstains in a reliable and quantifiable manner. Much less information is derived from knowing precisely what type of fly artifact is present.

The composition of defecatory stain offers insight into the possible development of new presumptive and confirmatory tests to detect fly fecal stains. Uric acid and allantoin are present in the excreta of adult and larval flies, although the concentration varies based on developmental stage [28,33]. In the absence of hyperuricemia condition, human blood contains only trace amounts of uric acid, and humans are incapable of naturally synthesizing allantoin [62,63]. Thus, like occurs with acid phosphatase in presumptive testing of human semen [64], concentration (high levels) differences of uric acid in irregular shaped ‘bloodstains’ may be indicative of defecatory stains. Similarly, allantoin detection through enzymatic assays [65] could potentially be developed into a confirmatory test for fly defecate.

### 4.2. Molecular Methods

The development of molecular methods for identifying fly artifacts would allow for more conclusive determination of artifacts than most current methods. Design of these methods will likely depend on identification of DNA sequences present in the artifacts, rather than RNA or protein as these macromolecules are more difficult to detect in extremely small quantities and require additional manipulation before analysis. Since human blood stains, as well as fly regurgitate and defecatory stains could all have human DNA present, distinguishing between these types of artifacts at a crime scene would benefit from being able to identify insect DNA within artifacts. Fly gut epithelial cells are routinely shed over the lifespan of the organism leading to frequent intestinal regeneration [66,67]. While much of this research comes from the study of *Drosophila melanogaster* [68,69,70], there is a high likelihood that this developmental process occurs with many species of necrophagous Diptera. Research into the existence of epithelial cells shed from the gut in either regurgitate or excrement, could determine the presence of insect DNA in these artifacts. Identification of this insect DNA could be performed by looking for high copy number DNA sequences within the fly genome following genomic sequencing. The use of high copy number sequences, such as ribosomal genes and transposons, to make an organism determination would result in detection that is more feasible given the low number of cells likely present in an individual fly artifact. Additionally, quantitative polymerase chain reaction (qPCR) could be used to amplify insect DNA from artifacts as a means for organism detection. The use of degenerative primers for qPCR would allow for an assay in which the specific fly species does not need to be known prior to assay initiation, making the assay easy to use and less costly than trying primers for each specific fly species in an attempt to determine which flies were present.

### 4.3. Fly Microbiome

The endogenous microbiome of necrophagous flies is an attractive area of study to differentiate fly artifacts from human bloodstains. Investigation into the microbial flora of insects commonly found at crime scenes may lead to a catalog of microbes that could be identified within fly artifacts, and presumably absent from human bloodstains and other bodily fluids, at least at the time of death. Detection could be accomplished using DNA extraction followed by bacterial 16S rDNA tag encoded FLX-titanium amplicon pyrosequencing (bTEFAP), generating short DNA sequence reads [71,72]. These DNA sequences could then be classified by bacteria taxonomy based on sequence. Analysis of the normal microbiome of two calliphorids, *L. sericata* and *L. cuprina*, has been undertaken for various life stages [72]. The feasibility of obtaining enough microbial DNA from fly artifacts for analysis will need to be studied, but identification of even a low number of microbial sequences from adult flies common to a crime scene location may be enough to distinguish the spot from human bloodstains. With this type of analysis, the microbiome likely will change with timing of colonization and developmental stage for both for the fly and microbes. The anticipated result is deposition of endogenous fly microbes as well as microbes newly ingested from a decaying corpse in fly artifacts [73].

## 5. Conclusions

Necrophagous Diptera that colonize a corpse, carrion, or an animal or person with open wounds may confound crime scenes by altering body fluid stains. Both adults and larvae have the potential to distort existing body fluid stains or depositing contaminants in the form of artifacts that are morphologically similar in size, color and shape to human body fluids. Adult flies deposit regurgitate and defecatory stains released from opposing ends of the digestive tract. Meconium is released almost immediately upon emergence from puparia, but is typically restricted to locations near the site of extrication. Transfer patterns are produced as either translocation (from dragging body parts) or tarsal tracks, which are literally fly footprints. By contrast, crawling larvae can distort existing stains as well as yield transfer patterns. Additional larval artifacts can be derived from the alimentary canal in the form of secretions or excretions. At present, fly artifact are detected based on morphological and contextual criteria, which are not consistently reliable in distinguishing insect stains from human body fluids. New research should focus on the development of diagnostic tools for distinguishing fly artifacts from human body stains based on the chemical composition of fly contaminants. This idea is based on the premise that each type of fly-derived fluid contains chemical constituents unique to the condition that produced them.

## Figures and Tables

**Table 1:** Morphological characteristics of fly artifacts.

Stain Type	Shape	Color	Dimensions *
Regurgitate			Area in mm^3^
	round to asymmetrically round, occasionally with small tails to form tear drop shape	highly variable and dependent on food source. May be clear, red, green, gray tan/light, dark brown/black	3.0–16.2 (human blood)
			1.9–19.2 (bovine blood)
Defecatory			Tail length (mm)
	round to asymmetrically round, some possessing long tails that form tad pole, tear-drop, and sperm-like shapes	highly variable and dependent on food source. May appear creamy, dark brown/black, gray, tan/light ^#^	3.0–16.2 (human blood)
			1.9–19.2 (bovine blood)
			4.8–9.2 (chicken blood) ^+^
Translocation			Stain length (mm)
	asymmetrically linear	same as food source	0.98–10.4 (human blood)
			0.74–9.7 (bovine blood)
Tarsal tracks	small and round, impressions of tarsi or pulvilli	same as food source	>0.2 mm in diameter

* Data from [12] using *Sarcophaga bullata* (Sarcophagidae), *Calliphora* *vicina* (Calliphoridae), *Chrysomya rufifacies*, *Ch. megacephala*, and *Phormia regina*. ^+^ Data from [20] using *Ch. megacephala* feed chicken blood. ^#^ Data from [19] using *Lucilia cuprina* fed human blood.
